# Early-life begging effort reduces adult body mass but strengthens behavioural defence of the rate of energy intake in European starlings

**DOI:** 10.1098/rsos.171918

**Published:** 2018-05-09

**Authors:** Jonathon Dunn, Clare Andrews, Daniel Nettle, Melissa Bateson

**Affiliations:** Centre for Behaviour and Evolution, Institute of Neuroscience, Newcastle University, Newcastle, UK

**Keywords:** energy intake, early-life adversity, foraging behaviour, *Sturnus vulgaris*, begging, body mass

## Abstract

Animals require strategies for coping with periods when food is scarce. Such strategies include storing fat as a buffer, and defending the rate of energy intake by changing foraging behaviour when food becomes difficult to obtain. Storage and behavioural defence may constitute alternative strategies for solving the same problem. We would thus expect any developmental influences that limit fat storage in adulthood to also induce a compensatory alteration in adult foraging behaviour, specifically when food is hard to obtain. In a cohort of hand-reared European starlings, we found that higher manipulated early-life begging effort caused individuals to maintain consistently lower adult body mass over a period of two years. Using an operant foraging task in which we systematically varied the costs of obtaining food, we show that higher early-life begging effort also caused stronger behavioural defence of the rate of energy intake when food was more costly to obtain. Among individuals with the same developmental history, however, those individuals who defended their rate of energy intake most strongly were also the heaviest. Our results are relevant to understanding why there are marked differences in body weight and foraging behaviour even among individuals inhabiting the same environment.

## 1. Introduction

Food availability often varies in the wild; sometimes food is abundant, whereas at other times it is scarce and more difficult to obtain. Animals have evolved a range of strategies for avoiding starvation in the face of food scarcity. These strategies can be broadly divided into: carrying high energy reserves in the form of fat to provide a buffer (henceforth storage); and temporary behavioural responses to defend the rate of energy intake during periods of food scarcity (henceforth defence). A number of different defence mechanisms have been identified, including: expanding the range of foraging locations or food types, increasing toxin tolerance, and simply increasing foraging effort. Studies often document substantial within-population variation in body mass, even under the same environmental conditions [1–3], and also in appetitive and consummatory food motivation, even among individuals that are food-deprived to the same extent [4–6]. Thus, there appear to be individual differences in the extent to which storage and defence are deployed.

What should be the relationship between an individual's level of storage and its level of defence? Carrying more body fat and increasing defence of food intake both decrease starvation risk but may increase predation risk, resulting in a trade-off between risk of predation and risk of starvation. From an adaptive point of view, animals should minimize the sum of their expected mortality from starvation and from predation [7,8]. Because of variation in overall quality, locomotor ability and other physiological parameters, different individuals should accept different levels of starvation risk. For example, an individual that is very good at avoiding predation at a given body mass or level of food intake defence should accept a lower starvation risk than an individual less good at avoiding predation. Given the predation–starvation trade-off, a lower-quality individual may have to accept greater starvation and predation risk than a higher-quality one. Because some individuals accept lower overall starvation risk than others, and because storage and defence are both strategies to reduce starvation risk, we expect to observe positive correlations between body weight and foraging-behaviour defence when we compare across individuals from unselected populations. Indeed, positive associations between body weight and food motivation are commonly reported in both mammals and birds [9–11]; however, whether the behavioural tasks used in such studies really capture defence of the rate of energy intake, rather than just the rate of energy intake when food is easy to obtain, is not clear.

Within an individual, by contrast, there might be a compensatory relationship between storage and defence. An individual that cannot currently store much fat must upregulate its defence strategies in order to achieve any given risk of starvation. Thus, comparing the same individual across states where it had different levels of stored reserves, we would expect to observe a negative relationship: assuming an animal has a fixed predation risk it is willing to accept, as stored reserves decrease, behavioural defence of the rate of energy intake should become stronger. In support of this claim, within-subjects studies find that food-restricted or lighter animals become more willing to forage in potentially dangerous places [12] and are less food selective [13].

Just as externally imposed food restriction in adulthood might evoke behavioural compensation, so developmental programming could lead to a long-term compensatory shift in the mix of storage and defence strategies. There is growing evidence that developmental experience affects fat storage in adulthood. For example, rodents raised in experimentally reduced litters, which receive more milk *per capita* and grow more quickly, maintain higher body masses than controls in adulthood, whereas rats raised in experimentally enlarged litters display the opposite pattern [14]. House sparrows exposed to corticosterone show reduced mass for size as adults [15]. If developmental influences such as these effectively constrain individuals from storing as much in the form of fat as they otherwise would, but without changing the balance point of starvation risk against predation risk, then we can make the following general prediction: any developmental influence that constrains the amount of fat reserves an individual carries as an adult should also influence the development of behavioural defence mechanisms to compensate. In effect, there would be compensatory plasticity: if an individual was canalized to be thin, it would plastically develop stronger behavioural defences against starvation. In uncontrolled population studies, this compensatory relationship is usually likely to be masked by the fact that individuals vary in other ways that affect the total level of starvation risk they must accept (though see [3] for an exception where the compensatory relationship was found). However, by experimentally exposing young animals to developmental influences that are likely to alter their fatness as adults, we can test whether their behavioural defence of the rate of energy intake is upregulated.

Some evidence from rodents suggests that individuals exposed to early-life undernutrition work harder to obtain food particularly when food is unpredictable or costly [16,17], and in a recent study of European starlings, we showed that adults that had been reared in large broods rejected fewer toxic but nutritious prey (when no other foods were available) than those reared in small broods [18]. This suggests developmental experience can shift individuals towards stronger behavioural defence of the rate of energy intake. What has not yet been established is whether the same developmental influences alter fat storage and defence in a correlated, compensatory way.

We investigated whether developmental influences that lead to lower body mass in adulthood also lead to stronger behavioural defence of the rate of energy intake, in a cohort of hand-reared European starlings in which we manipulated early-life feeding schedules. Starlings are a suitable study system because they are a wild species in which good measures of the natural variation in developmental experience are available [19–21]. They are plastic in both mass and behaviour, and have served as a model species for the analysis of adaptive control of energy reserves in response to variation in the adult environment [20,22,23]. Finally, in altricial birds such as the starling it is possible to rear by hand, and thus exert very strong control over early environmental inputs.

The classic manipulation of post-natal early-life experience in both rodents and birds is altering litter or brood size. While naturalistic, this manipulation has the disadvantage of altering several parameters simultaneously. In particular, in large litters or broods, individuals not only receive less food *per capita*, but also have to work harder to obtain it. This takes the form of begging and jostling in nestlings, and rooting, climbing and jostling in rodent pups [24,25]. Thus, it is not clear from brood- or litter-size manipulations what the key causal factor is. With this limitation in mind, we recently designed a hand-rearing manipulation [26] in which we independently varied, during 10 days of early life, the amount of food received (henceforth Amount; either Plenty or Lean), and the minutes of begging per day (henceforth Effort; either Hard or Easy). Both treatments appear to have affected adult behaviour, albeit in different ways [27,28]. Here, we investigate whether the developmental treatments affected body mass, and foraging effort in the face of increasing difficulty in obtaining food, in adulthood.

We tracked the birds' body masses over a period of two years, during which the birds were sometimes housed in flocks in aviaries, and sometimes in individual cages. We were thus able to examine the effects of the developmental treatments on mass, both in the presence and the absence of social competition. To assess behavioural defence of food intake, we tested the birds using an operant task called a cyclic ratio schedule [29,30]. In this task, birds were required to forage by pecking a key, and the number of pecks required per reward (the ratio requirement) was cyclically increased and decreased. The cyclic ratio task thus allowed us to measure how strongly each individual defended its rate of energy intake as the cost of foraging changed.

## 2. Material and methods

### 2.1. Subjects

Our subjects were 32 hand-reared European starlings (16 male, 16 female) that were removed from nest-boxes in the wild on day 5 post-hatching in 2014. They comprised eight families of four siblings matched for weight on day 5. They were then subjected to a 10-day developmental manipulation (full details in [26]). Briefly, the Plenty groups received nine feeds to satiation per day. On each feeding visit, the Lean groups received a percentage of the mean amount consumed by the corresponding Plenty groups on the most recent feed. This began at 70% but was adjusted over the 10 days, averaging 73% overall. The Easy groups' artificial nests were visited just for the nine feeds each day. The Hard groups received an additional nine visits, during which they were stimulated to beg for 2 min (approx. the duration of a feed), but without any food being delivered. Thus nestlings in the Hard groups begged for approximately 36 min per day, compared with 18 min in the Easy groups. The manipulation thus resulted in four developmental treatment groups made up of the factorial combinations of food Amount and begging Effort: Plenty Easy (PE), Plenty Hard (PH), Lean Easy (LE), Lean Hard (LH). Unfortunately, we were not able to genetically sex our nestlings prior to commencing the developmental treatment, which led to an unbalanced sex ratio (M : F) for the LE (8 : 0) and PH (1 : 7) treatment groups. After day 15 post-hatching, the birds were all fed to satiation on every feed until fledging (around day 21). Following fledging, the birds were kept in mixed-treatment cages with ad libitum food until they had all been observed feeding themselves, and then released into two indoor aviaries (215 × 340 × 220 cm; approximately 18°C, 40% humidity; 15 L : 9 D light cycle) in mixed-sex groups of no more than 20 birds on day 56 post-hatching, again with ad libitum food. The birds were maintained in non-breeding condition by the light cycle.

Over the first two years of life, the birds lived part of the time in aviaries in mixed-treatment groups, and part in individual cages for behavioural experiments. Individual cages measured 100 × 45 × 45 cm, with two perches and plastic baths, and the same light cycle as the aviaries. Ad libitum cat biscuits (Royal Canin Ltd. ‘Fit'), Orlux insect paté, domestic chick crumb (Special Diets Services ‘Poultry Starter (HPS)'), fresh fruit and live mealworms were given to the birds housed in aviaries. Birds housed in cages received ad libitum domestic chick crumb and a limited number of live mealworms per day, depending on the exact nature of the experimental protocol.

### 2.2. Body masses

Body mass measurements were collected over a period of 757 days (from 20 June 2014 on post-hatch day 56 until 16 July 2016) prior to the cyclic ratio task. The birds were weighed as part of periodic routine health checks, whenever they were removed from the aviaries to begin behavioural experiments, and again during those behavioural experiments. All masses were collected between 15.00 and 17.00 using a weighing cone and digital scales (Oertling GC62 accurate to 2 d.p.). Although the time of day that the birds were weighed was approximately the same, the dates on which the birds were weighed were not evenly distributed across the data collection period and nor were they on the same day for every bird. There were 32 birds at the start of the data collection period, but two birds died before they were 20 months old (one Lean Easy bird died in an accident and one Lean Hard bird died due to unknown causes). All masses collected up until the point of death were retained in the analysis, as they were not thought to reflect illness or any other fundamental change to underlying state. One weight measurement was excluded, where illness was suspected to have caused an uncharacteristically low value. The dataset analysed thus comprised 698 mass measurements (mean ± s.d. = 22 ± 4 measurements per bird). We also measured tarsus length using digital callipers at the end of the developmental period (day 56) and used the mean of two replicate measurements of each leg.

### 2.3. Operant task

The cyclic ratio (henceforth CR) task was completed when birds were approximately two years of age (starting 4 May 2016). Birds were moved to individual operant cages in groups, keeping natal families together and hence balancing testing order across developmental treatments. Each cage was fitted with a panel consisting of three illuminable pecking keys and a feeder trough connected to a pellet dispenser delivering 45 mg grain-based rodent pellets (TestDiet, Richmond IN, USA) (our experimental set-up is fully described elsewhere [31], though note that study used different birds). Experimenters were never present in the experimental room during testing, as operant panels were controlled by a remote computer using the Whisker Experimental Control system [32]. Behavioural tasks were programmed in Microsoft Visual Basic 6.0 (Microsoft Corporation, Redmond, WA, USA). The CR task reported here followed immediately after another operant task addressing a different question. Hence, the birds were already habituated to being in cages and were trained to peck keys for pellets [31].

The CR task comprised a discrete-trials ratio schedule in which the ratio requirement in each trial changed cyclically within each session. A trial began with illumination of the centre key in amber. The bird was required to peck the lit key a number of times specified by the current ratio requirement. Successful completion of this requirement within 1800 s resulted in the key light extinguishing, release of 1 food pellet and illumination of the feeding trough for 3 s. Failure to complete the requirement within 1800 s resulted in the key light extinguishing and the trial ending. All trials ended with an inter-trial interval of 1 s. The sequence of ratio requirements used was: 2, 4, 6, 8, 10, 12, 12, 10, 8, 6, 4, 2; and this cycle was repeated up to five times within each session (maximum of 3 h). We chose an arithmetic progression of ratios to address concerns that birds might fail to complete very high ratios [33]. The schedule parameters were chosen to ensure the birds did not become satiated [34,35]. Operant sessions began at 07.00 (1 h after lights coming on). Ad libitum food (domestic chick crumb) and baths were provided at 10.00. These were removed at 17.00 in preparation for the following morning's session. Birds thus had 14 h of food deprivation (including the night) prior to each operant session. Each bird was exposed to one daily session of the CR task for two successive days resulting in a maximum of 120 trials per bird. The latency between the first and final key peck was collected for every trial and is hereafter termed ‘trial latency'.

### 2.4. Data analysis

All data analyses were undertaken in R v. 3.3.2 [36]. The raw data files and the R script are available at the Zenodo repository [37]. We fitted linear mixed models using the package lme4 [38]. For the main analyses, we employed model selection and model averaging procedures following Grueber *et al.* [39]. We first centred and standardized our predictors on 2 s.d. [40] using the ‘arm' package [41]. We used the MuMIn package [42] to create and rank models with all possible combinations of the candidate fixed effects (detailed below) using Aikaike's corrected information criterion (AICc) [43]. Those models that were within two AICc of the lowest value were retained as a best-models subset [44], which were then averaged to obtain parameter estimates and their confidence intervals. To evaluate variance explained, we calculated the marginal *R*^2^, $R_{\mathrm{LMM}(m)}^{2}$, which is the variance explained by the fixed factors in the model, and the conditional *R*^2^, $R_{\mathrm{LMM}(c)}^{2}$, which is the variance explained by both the fixed and random factors [45]. All models presented here gave satisfactory distribution of residuals, hence a Gaussian error structure was assumed throughout, and variance inflation factors of less than 2. Single-term models (not shown) produced parameter estimates that resembled the sign, size and significance of those obtained from model averaging.

For body mass as a response variable, candidate fixed effects were age (continuous), sex (M/F), tarsus length (continuous), housing type (aviary/individual cage), food Amount (Lean/Plenty) and begging Effort (Hard/Easy), with random effects of natal nest and individual identity due to the repeated masses from the same individuals within families. The mass model also included all possible interactions between Amount, Effort, age and housing type. By including tarsus length in our models, we controlled for individual differences in skeletal size [46].

For the CR task, the unit of analysis was the trial, and the response variable was the logged trial latency. Candidate fixed effects were: sex, tarsus length, ratio requirement, Amount, Effort and all possible interactions between Amount, Effort and ratio requirement. Random effects were natal nest and individual identity. Stronger defence of energy intake as food becomes harder to obtain will manifest as a shallower slope of increasing trial latency with increasing ratio requirement. Thus, if any predictor variable is associated with stronger defence, this will appear as an interaction between that predictor and ratio requirement. Within the CR task, defence of energy intake is often measured as the gradient of the negative slope (the ‘Staddon slope') of the association between response rate (which is the ratio divided by the trial latency) and feeding rate (the reciprocal of the trial latency) [29]. This measure is problematic as the trial latency appears on both sides of the linear equation, and not all individuals show negative slopes, so we did not use Staddon slopes in our main analyses. However, in the electronic supplementary Material, §S1 and figure S1, we show that, for those birds for which both our slope (trial latency against ratio requirement) and the Staddon slope (response rate against feeding rate) are defined, the two slopes are correlated at *r* = 0.67.

Given that sex ratio was unbalanced by the treatment group, we also constructed models that included significant parameters and interactions retained by our model selection and averaging procedures, and additionally included interactions between sex : Amount and sex : Effort. This was to rule out the possibility that our results reflected differences in sex, instead of the intended effect of developmental treatment. The inclusion of these two additional interactions did not change our results in any meaningful way, nor did they themselves have a significant effect on our outcome variables. Consequently, we judged it unlikely that the bias in sex ratio confounded the effects of developmental treatment that we report subsequently.

Finally, to examine the association between individuals' overall mean masses and their defence of the rate of energy intake, we calculated a single-value measure of defence for each individual in the form of the slope of logged trial latency against ratio requirement for each bird (henceforth, the latency slope), and tested for associations with individual mean body mass over the entire time series, after controlling for tarsus length. We did not undertake model selection or model averaging for this simple analysis.

## 3. Results

### 3.1. Body masses

We examined how body masses over time were affected by the developmental treatments, after controlling for skeletal size. The global model explained around 68% of the variation in body mass $\left(R_{\mathrm{LMM}(c)}^{2}=0.68\right)$ of which around 46% was explained by fixed factors and their interactions $\left(R_{\mathrm{LMM}(m)}^{2}=0.46\right)$. Two top models were retained for model averaging (table 1) with the results suggesting that body mass was best explained by a combination of main effects of housing, sex, tarsus length, age and Effort; and interactions between housing and Effort, and housing and age (table 2).

**Table 1.** Best-model subsets for body mass and trial latency from the operant cyclic ratio task. AICc refers to Akaike's information criterion corrected for small sample sizes; ΔAICc refers to the difference in AICc between each model and the model with the lowest AICc in the subset; AICc wt refers to the AICc weight; bird refers to individual identity; ratio refers to ratio requirement.

response variable	random effects	subset model no.	fixed effects	AICc	ΔAICc	AICc wt	log likelihood
body mass	natal nest/bird	1	Sex + Tarsus + Age + Housing + Effort + Housing : Effort + Housing : Age	4178.80	0.00	0.73	−2078.21
		2	Sex + Tarsus + Age + Housing + Effort + Housing : Effort + Housing : Age + Age : Effort	4180.80	1.99	0.27	−2078.17
trial latency	natal nest/bird	1	Amount + Effort + Ratio + Effort : Ratio	13 793.60	0.00	0.26	−6888.79
		2	Amount + Sex + Effort + Ratio + Effort : Ratio	13 794.40	0.74	0.18	−6888.15
		3	Amount + Effort + Ratio + Amount : Ratio + Effort : Ratio	13 794.80	1.19	0.14	−6888.38
		4	Effort + Ratio + Effort : Ratio	13 795.30	1.68	0.11	−6890.63
		5	Amount + Effort + Ratio + Tarsus + Effort : Ratio	13 795.50	1.86	0.10	−6888.71
		6	Amount + Sex + Effort + Ratio + Amount : Ratio + Effort : Ratio	13 795.50	1.94	0.10	−6887.74
		7	Amount + Effort + Ratio + Amount : Effort + Effort : ratio	13 795.60	1.95	0.10	−6888.75

**Table 2.** Model averaged parameter estimates for predictors of body mass and trial latency in the operant cyclic ratio task. Predictors were standardized on 2 s.d.

response variable	random effects	fixed effects	estimate	s.e.	CI 2.5%	CI 97.5%	relative importance
body mass	natal nest/bird	Effort_Easy	2.02	1.28	−0.50	4.53	1.00
		Housing_Cage	−9.42	0.39	−10.18	−8.66	1.00
		Sex_Male	3.36	1.37	0.73	5.98	1.00
		Tarsus	4.06	1.14	1.83	6.29	1.00
		Age	2.96	0.38	2.21	3.70	1.00
		Housing_Cage : Effort_Easy	−2.18	0.77	−3.69	−0.67	1.00
		Housing_Cage : Age	−3.93	0.78	−5.46	−2.39	1.00
		Effort_Easy : Age	0.21	0.76	−1.27	1.70	0.27
log(trial latency)	natal nest/bird	Amount_Plenty	−0.39	0.19	−0.76	−0.01	0.89
		Effort_Easy	−0.16	0.19	−0.54	0.21	1.00
		Ratio	0.58	0.05	0.48	0.69	1.00
		Effort_Easy : Ratio	0.26	0.12	0.05	0.48	1.00
		Sex_Male	−0.25	0.22	−0.68	0.18	0.28
		Amount_Easy : Ratio	−0.10	0.11	−0.31	0.11	0.24
		Tarsus	0.08	0.35	−0.34	0.51	0.10
		Amount_Plenty : Effort_Easy	−0.09	0.35	−0.78	0.61	0.10

**Figure 1.**
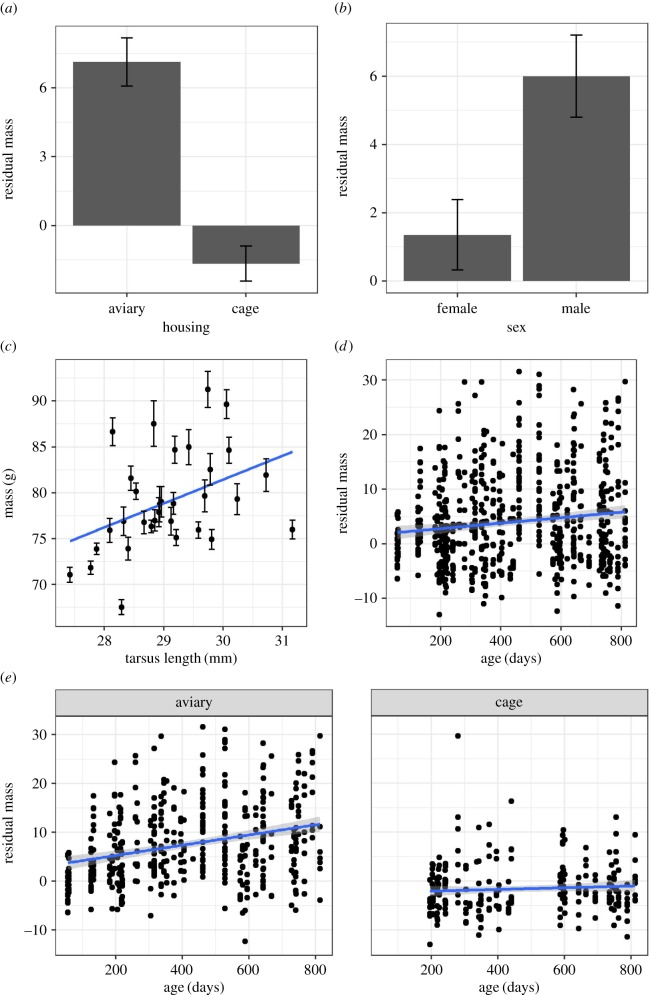
Body mass data split by covariates. (*a*) Individual mean residual body mass (after controlling for day 56 tarsus length) and between-bird s.e. by housing type. (*b*) Individual mean residual body mass (after controlling for day 56 tarsus length) and between-bird s.e. by sex. (*c*) Scatter plot of individual mean body mass (plus within-bird s.e.) on day 56 tarsus length. Regression line represents a simple linear fit. (*d*) Scatter plot of residual body mass on day 56 tarsus length. Regression line represents a simple linear fit plus s.e. (*e*) Same as (*d*) but by the two levels of housing type. All panels are based on raw data from 32 birds.

Figure 1 shows the factors other than developmental treatments that affected body mass. Birds were heavier when housed in aviaries versus cages (*β*(cage) = −9.42, 95% CI from −10.18 to −8.66) and, unsurprisingly, birds that were skeletally larger were also heavier (*β* = 4.06, 95% CI from 1.83 to 6.29). Male birds were consistently heavier than female birds (*β*(male) = 3.36, 95% CI from 0.73 to 5.98) and birds also got heavier with age (*β* = 2.96, 95% CI from 2.21 to 3.70), although the latter effect was more pronounced when the birds were housed in aviaries when compared with cages (*β*(cage:age) = −3.93, 95% CI from −5.46 to −2.39).

**Figure 2.**
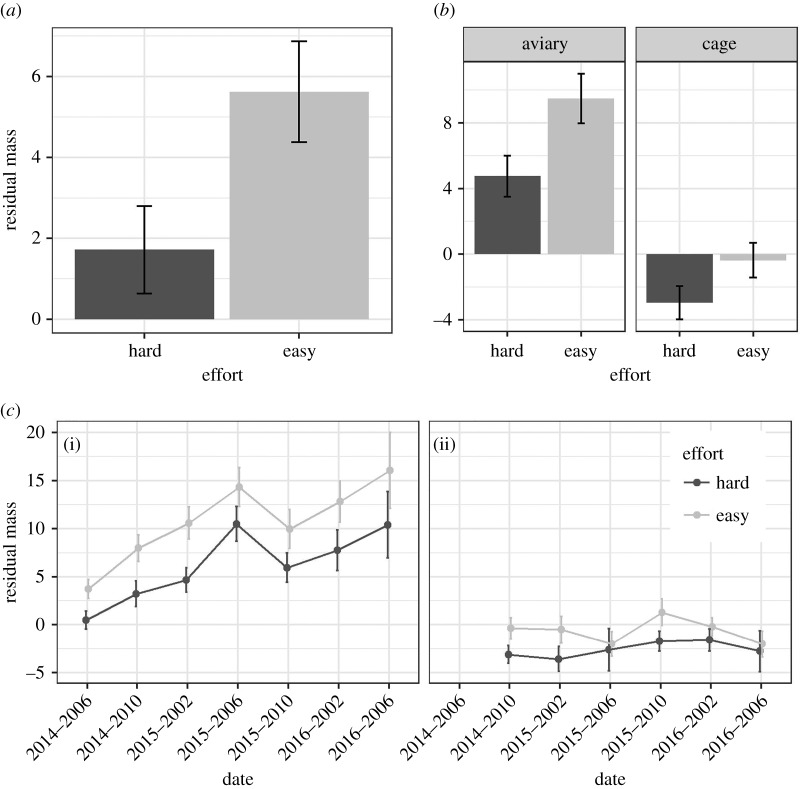
Body mass data split by begging Effort developmental treatment. (*a*) Individual mean and between-bird s.e. of residual body mass (after controlling for day 56 tarsus length) by the two levels of the begging Effort developmental treatment. (*b*) Same as (*a*) but also divided by the two types of housing. (*c*) Individual mean and between-bird s.e. of residual body masses by date, pooled into four-month time bins, with aviary masses in (i) and individual cage masses in (ii). Masses are from fledging in June 2014 onwards. All panels are based on raw data from 32 birds (16 Hard and 16 Easy birds).

For the developmental treatments, there was weak evidence that Easy Effort birds were heavier for their skeletal size than Hard Effort birds overall (*β*(Easy) = 2.02, 95% CI from −0.50 to 4.53; figure 2), but strong evidence for the effect when the birds were housed in aviaries rather than cages (*β*(Easy:cage) = −2.18, 95% from −3.69 to −0.67; figure 2).

### 3.2. Foraging behaviour

We examined how the logged latency to complete the trial in the CR task responded to variation in the ratio requirement, in interaction with our developmental treatments. The global model only explained around 12% of the variation $\left(R_{\mathrm{LMM}(c)}^{2}=0.12\right)$ and of this 5% was explained by fixed factors and their interactions $\left(R_{\mathrm{LMM}(m)}^{2}=0.05\right)$. Seven top models were retained for model averaging (table 1). Within this set, high relative importance was given to Amount, Effort, ratio requirement, and the interaction between Effort and ratio requirement (table 2). Sex, tarsus length and the interactions between Amount and ratio requirement, and Amount and Effort featured in the best-model set but with low relative importance and parameter estimates whose 95% CI included zero.

**Figure 3.**
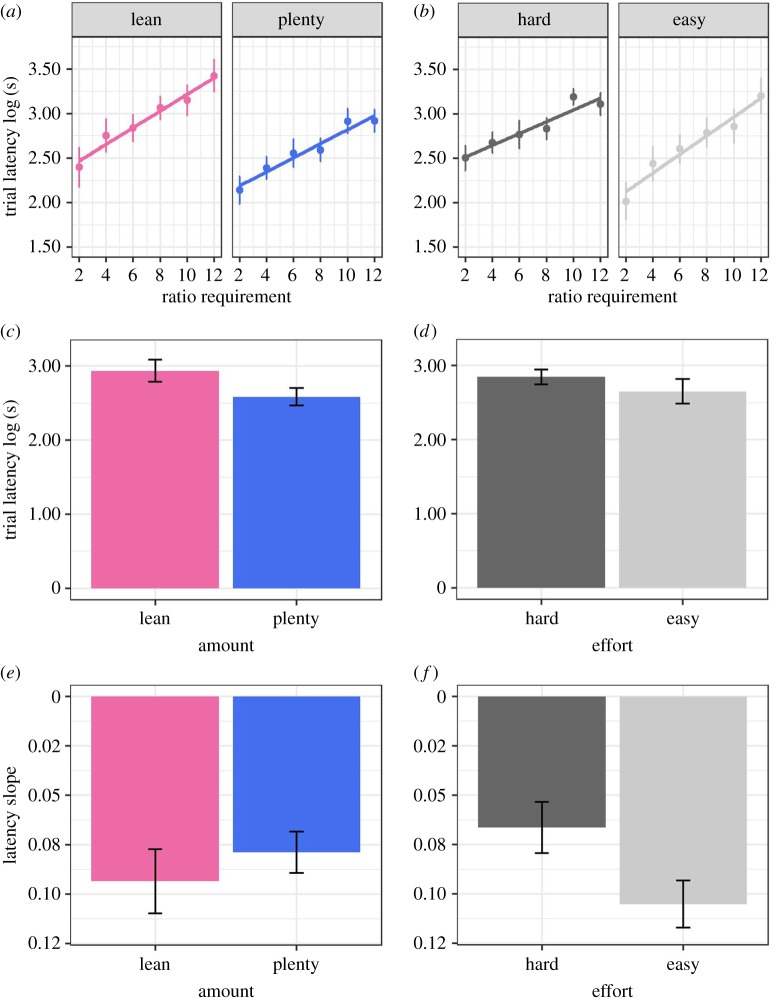
Behavioural data: trial latencies and latency slopes. (*a*) Individual mean and between-bird s.e. of trial latency by ratio requirement (pecks required to complete a trial), and by the two levels of the Amount developmental treatment. (*b*) Same as (*a*) but split by the two levels of the Effort developmental treatment. (*c*) Mean individual trial latency plus between-bird s.e. by the two levels of the Amount developmental treatment group. All ratio requirements are pooled. (*d*) Same as (*c*) but split by the two levels of the Effort developmental treatment; (*e*) Mean latency slope (the slope of the individual relationship of average trial latency against ratio requirement, plus between-bird s.e.) by the two levels of the Amount developmental treatment. Note that the *y*-axis is inverted so that a higher position reflects stronger defence. (*f*) Same as (*e*) but divided by the two levels of the Effort developmental treatment. Panels are based on data from 30 birds (14 Lean and 16 Plenty birds in panels *a*, *c* and *e*; and 15 Hard and 15 Easy birds in panels *b*, *d* and *f*).

Plenty Amount birds had lower trial latencies than Lean Amount birds overall (*β*(Plenty) = −0.39, 95% CI from −0.77 to −0.01; figure 3). Thus, birds that had experienced early-life food restriction completed trials more slowly on average than those that had not. Hard Effort birds had a shallower increase in latency as ratio requirement increased than Easy Effort birds (*β*(Hard × ratio requirement) = −0.26, 95% CI from −0.48 to −0.05; figure 3). Thus, Hard Effort birds showed better defence of their rate of food intake as the effort required to obtain food increased.

### 3.3. Relationship between body mass and defence of rate of food intake

**Figure 4.**
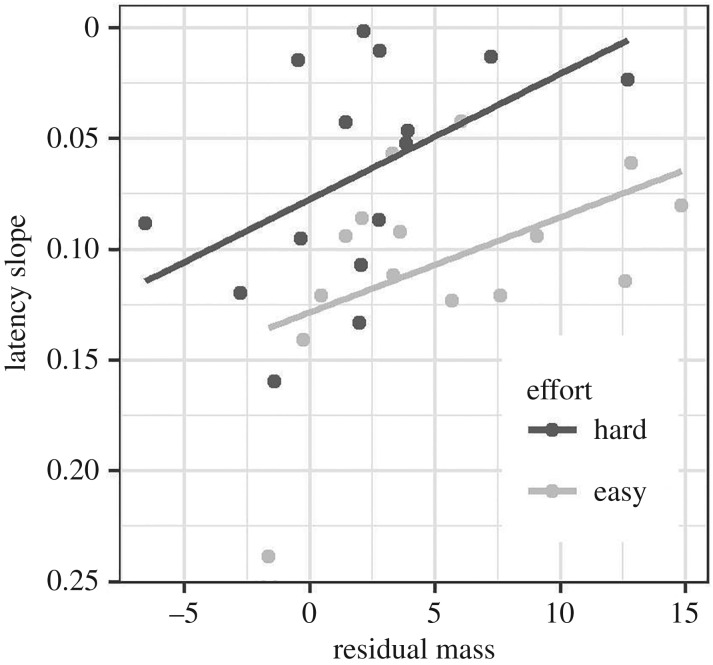
Relationship between body mass and defence of energy intake. Scatter plot of individual latency slope (inverted so that a higher position represents stronger defence) against the individual's residual body mass over the whole mass time series (after controlling for day 56 tarsus length). Lines represent simple linear fits for each group. Panel based on data from 30 birds (15 Hard and 15 Easy birds).

We plotted the latency slope for each bird against average residual body mass (i.e. the average of body mass after controlling for tarsus length, over the entire time series), splitting the dataset by level of Effort (figure 4). In accordance with the previous analysis, latency slopes were higher (i.e. defence was weaker) for the Easy birds (*β*(Easy) = 0.06, 95% CI from 0.02 to 0.09; table 3). However, after controlling for Effort, greater body mass was associated with lower latency slope, and hence stronger defence (*β*(body mass) = −0.05, 95% CI from −0.09 to −0.02; table 3). Thus, within each Effort group, the individuals that defended their feeding rate most strongly were the heaviest for their skeletal size, even though the group difference was in the opposite direction (the group with the stronger average defence was lighter on average).

**Table 3.** Linear mixed model parameter estimates for predictors of latency slope. Predictors were standardized on 2 s.d.

response variable	random effects	fixed effects	estimate	s.e.	CI 2.5%	CI 97.5%
latency slope	natal nest	Body mass	−0.05	0.02	−0.09	−0.02
		Tarsus	0.02	0.02	−0.01	0.01
		Effort_Easy	0.06	0.02	0.02	0.09
		Body mass : Effort_Easy	0.02	0.04	−0.05	0.09

## 4. Discussion

We examined how early-life experiences of food restriction (Lean Amount) or high begging effort (Hard Effort) affected body mass and behavioural defence of the rate of food intake in adulthood in a cohort of European starlings. When living in aviaries, the birds that had experienced the Hard (high begging effort) treatment carried a size-corrected body mass of around 2 g less than birds who received the Easy (low begging effort) treatment, and this difference showed no sign of attenuating after two years. The difference between the treatments was much less marked when the birds were in individual cages, where masses were substantially lower overall. Birds that had received the Hard begging effort treatment also defended their rate of energy intake more strongly, as evidenced by a slower increase in latency to gain a food reward as the cost of foraging (number of pecks required) was increased. Furthermore, these birds also foraged less rapidly in comparison with birds that had received the Easy treatment when the costs of obtaining food were low. Thus, the developmental manipulation appears to have canalized the birds into one group (Hard Effort) that remained lean but used their behaviour to defend their energy intake when food was costly to obtain, and another group (Easy Effort) that carried greater mass and allowed their rate of energy intake to drop when food became costly. The effects of developmental treatment are quite striking given that every Hard bird had a sibling in the corresponding Easy group; that the developmental manipulation only lasted ten days; and that the adult living conditions were uniform.

We found a different relationship between body mass and the strength of the behavioural defence of the rate of energy intake within and between developmental treatments. Comparing treatments, the treatment that was relatively heavier (Easy Effort) defended less strongly. Comparing individuals within the same treatment, however, there were positive associations: those individuals that defended most strongly were also the heaviest within the treatment. Our interpretation of this apparently paradoxical relationship is the following: non-treatment factors determined where on the trade-off curve between starvation and predation each individual sat. Thus, within a treatment, we are comparing individuals who accept different total risks of starvation; and in order to achieve lower total risks of starvation, some individuals do *both* more storage of reserves and stronger behavioural defence than others. The begging Effort treatment, however, appears to alter the mix of storage and defence deployed *at a given level* of overall starvation risk, with the Hard birds retaining the relative leanness that began in early life, and hence compensating with stronger behavioural defence. Whether the relative leanness represents a constraint imposed by developmental conditions, or is itself an adaptive strategy, is not clear. One possibility is that early-life adversity specifically impairs take-off ability as mass increases, a possibility for which there is some empirical evidence [47,48]. In this case, storage of reserves would be a more costly strategy relative to behavioural defence for Hard birds, and hence the optimal mix of storage and defence would be shifted in the direction of stronger defence. These findings reinforce the general point that when studying individual differences in populations, positive correlations between phenotypic traits are often observed, but experimental manipulations can reveal trade-offs or compensatory negative relationships between traits [49].

Our findings are consistent with some aspects of our previous observations on these same birds, but inconsistent with others. During early development, it was the cross-factored Amount treatment (food restriction versus feeding to satiation) that most strongly affected mass gain [26], and yet in adulthood we found no systematic mass difference between birds that had experienced food restriction (Lean) and those that were fed to satiation (Plenty). We also observed no effect of the Amount treatment on defence of the rate of energy intake, though the Plenty birds were quicker overall than the Lean birds to peck keys for food. In a separate behavioural experiment, we found evidence that both treatments affected contrast effects when foraging, and in that experiment we incidentally observed that Hard birds were quicker than Easy to remove a heavy weighted lid (weighing over half the bird's body mass) in order to access a food reward [28]. This is in effect an independent measure of defence of feeding rate when food is difficult to obtain, and hence is consistent with our behavioural findings here. We have also found that the Hard birds from this cohort perch higher from the ground than the Easy birds [27]; this might corroborate our suggestion that take-off ability is particularly affected by the Effort developmental treatment.

Comparing our findings to previous literature, the fact that Lean Amount did not affect adult body mass concurs with several other avian studies [50–52] showing that early food restriction is compensated for in adult mass (though see [53] for a divergent finding). The long-term effect of Effort on adult mass is reminiscent of the rodent litter-size literature, where pups from large litters, and hence who have to compete more strongly to be fed, maintain consistently lower body masses in adulthood [14]. Our finding of stronger behavioural defence of the rate of energy intake in the Hard treatment is partially consistent with the rodent literature showing an impact of rearing conditions on foraging rate particularly when food delivery is unpredictable or costly [16,17], though these studies manipulated something closer to our Amount treatment than our Effort treatment. It is also consistent with our previous observations in the starling that early-life adversity produces adults that are more willing to consume toxic prey [18], forage with greater efficiency and forage more for information about food [54]. The discrepancy is that in neither of these previous two studies were the early-adversity birds lighter for their skeletal size than the other group: in one study [18] there was no significant difference, and in the other [54] the early-adversity birds were significantly heavier. The early-life manipulations were different across all three of these studies: brood size [18] and position in the brood hierarchy [54] in our previous studies, and artificially induced begging effort in the present study. A possible explanation for the discrepancy in effects on adult body mass is that each of the three manipulations had different consequences for adult parameters such as overall quality and the starvation–predation trade-off relationship. We also note that in the two earlier studies, the masses were taken in the cages, whereas the clearest evidence for a mass difference between developmental treatments in the present study was when the birds were in aviaries.

Aside from our findings on developmental treatment, we found that all birds maintained lower body weights when housed singly in cages as opposed to aviaries. This difference was over 9 g on average, or more than 10% of body mass. The behavioural experiments performed in the individual cages typically involved several hours of food deprivation or food restriction during each day; however, in starlings and other passerines, periodic food unavailability leads to increased, not reduced, body mass, as birds store more fat as a buffer [22,23]. We suggest that the reduced body masses in individual cages may represent a response to lower competition or increased perceived predation risk. In aviaries, although we provide multiple bowls and ad lib food, the presence of a large number of conspecifics in a small space may induce the perception that competition could intermittently limit food access, provoking storage of a buffer. Previous experiments have shown that birds reduce body mass when exposed to predator cues [55]. Although our cage room contained no direct predator cues, due to the cage height, the birds were unable to perch more than around 30 cm from the ground. The birds were also caught more frequently in cages for welfare checks, possibly simulating predation attempts. Additionally, there was no possible dilution of perceived risk by perching close to conspecifics. It may thus be that the starlings perceived the cage environment as more dangerous than the aviaries, and hence maintained a lower body mass.

Strengths of our study were: the highly controlled manipulation of early-life experience via hand-rearing; the use of siblings to control for genetic effects; the uniform post-fledging conditions; the long follow-up period for studying adult body mass; and the precise characterization of adult foraging afforded by the operant cyclic ratio task. In particular, this task allows the defence of the feeding rate as the cost of foraging increases to be dissociated from the level of motivation when food is cheap [29,30]. A key limitation was that we did not measure fat reserves directly, but rather estimated storage of reserves from size-corrected body mass. However, in birds, variation in size-corrected body mass primarily reflects variation in fat stores [56]. In starling fledglings, for example, around 66% of the variance in size-corrected body mass was explained by lipid content established post-mortem [57]. We also have not measured metabolic rate, or general activity level: either of these could be affected by early-life experience [58], and both affect energy balance.

In animal ecology, there has been increasing awareness of the importance of individual differences in behaviour and physiology within populations [59], including individual differences in habitat selection [60]. The existence of such individual differences potentially affects both ecological and evolutionary dynamics [61]. While much of the focus has been on genetic sources of individual differences, here we have shown that variation in early developmental experience can generate alternative tactics for coping with food scarcity: fatness versus behavioural defence. Thus, individuals with different developmental histories may use their habitat in different ways, and may respond differently to environmental change [61]. This principle is also relevant to the human response to a changing food environment. As societies shift to industrial food production and a Westernized diet, rates of obesity increase markedly, but not all individuals become fatter to the same extent [62]. The way individuals respond to the food environment of adulthood may, in part, relate to childhood experience [63,64]. Thus, our findings in the starling may be relevant, at least as a proof of principle, to a broad range of species and problems.

## Supplementary Material

Supporting online materials for ‘Early-life begging effort reduces adult body mass but strengthens behavioural defence of the rate of energy intake in European starlings’

## Data accessibility

The raw data and R script from this experiment are publicly available at: (http://dx.doi.org/10.5281/zenodo.1034082) [37].
