# Supplementary material for: Early-life begging effort reduces adult body mass but strengthens behavioural defence of the rate of energy intake in European starlings
Source: R Soc Open Sci. 2018 May 9;5(5):171918. doi: 10.1098/rsos.171918 (PMC5990846; doi:10.1098/rsos.171918)
Supplement: Supporting online materials for ‘Early-life begging effort reduces adult body mass but strengthens behavioural defence of the rate of energy intake in European starlings’ [file rsos171918supp1.docx]

**Supporting online materials for ‘Early-life begging effort reduces adult body mass but strengthens behavioural defence of the rate of energy intake in European starlings’**

**1. Link between our behavioural measures and Staddon’s molar model of reinforcement**

Staddon’s molar model of reinforcement proposes that by plotting a straight line graph (termed a *response function*) of response rate against feeding rate for a range of ratio requirements, it is possible to calculate two independent measures of feeding behaviour (Staddon, 1980). The two measures are: i) the y-intercept of the response function, which indicates the subject’s preferred feeding rate and; ii) the slope of the response function, which indicates the subject’s willingness to defend its preferred feeding rate (sometimes termed *regulatory gain*). Note that these quantities are both related to the latency to complete the trial, since the response rate is the ratio requirement divided by the trial latency, and the feeding rate is the reciprocal of the trial latency.

Staddon’s measures are statistically problematic. The empirical variable collected, i.e. the latency to complete each trial, appears on both sides of the linear equation and hence both axes of the response function plot. Because the reciprocals of the latencies are used, the resulting parameter estimates have highly skewed distributions. Staddon assumes that individuals will exhibit partial but imperfect defence of preferred feeding rate, and hence that the response function will have a negative slope. If defence is perfect, the slope of the response function is infinite, and the y-intercept undefined. Individual animals sometimes show flat or positive response function slopes, and hence negative y-intercepts. The interpretation of the two measures for these individuals is not clear, and they have to be excluded from analysis.

Our approach as outlined in the main paper uses the latency data for each trial more directly, and results in interpretable parameter estimates for every individual. We reasoned that a higher preferred feeding rate would lead animals to be faster to complete trials at all ratio requirements. Thus, our measure of preferred feeding rate is simply the average logged trial latency in seconds across all trials regardless of their ratio requirement. Similarly, we reasoned that if an animal that defends its preferred feeding rate more strongly, its latency to complete the ratio will increase less steeply as the ratio requirement increases. Thus, our measure of defence of feeding rate is the slope of the relationship between the logged latency to complete the trial, and the ratio requirement (where stronger defence is indicated by a lower slope). Testing for the effect of a predictor on this parameter in a mixed model with trial as the unit of analysis equates to testing the significance of the interaction term between the predictor of interest and ratio requirement. For comparison between our analytical approach and the Staddon approach, we also calculated a single value per bird of the average trial latency (here using the median for comparability), and the slope of the relationship between trial latency and ratio requirement (fitting the line through the six median logged latencies corresponding to the six different ratios). We henceforth refer to this slope as defence of trial latency.

We verified that our measures were capturing the same constructs as Staddon’s original measures by calculating values for each bird for Staddon’s y-intercept and slope, and correlating these with our average trial latency and defence of trial latency. The number of birds that had negative response function slopes, and hence usable Staddon parameters, was 16. As required, there was a strong positive correlation between Staddon’s response function slope and our defence of latency slope (r_14_=0.67, p=0.002; Figure S1A), and a strong negative correlation between Staddon’s y-intercept and our average trial latency (r_14_=-0.73, p=0.0006 Figure S1B). This provides strong support for the idea that shorter average trial latencies captures substantially the same variation as higher response rates in the Staddon framework, and the slope of trial latency against ratio requirement captures substantially the same variation as the slope of the response function in the Staddon framework.


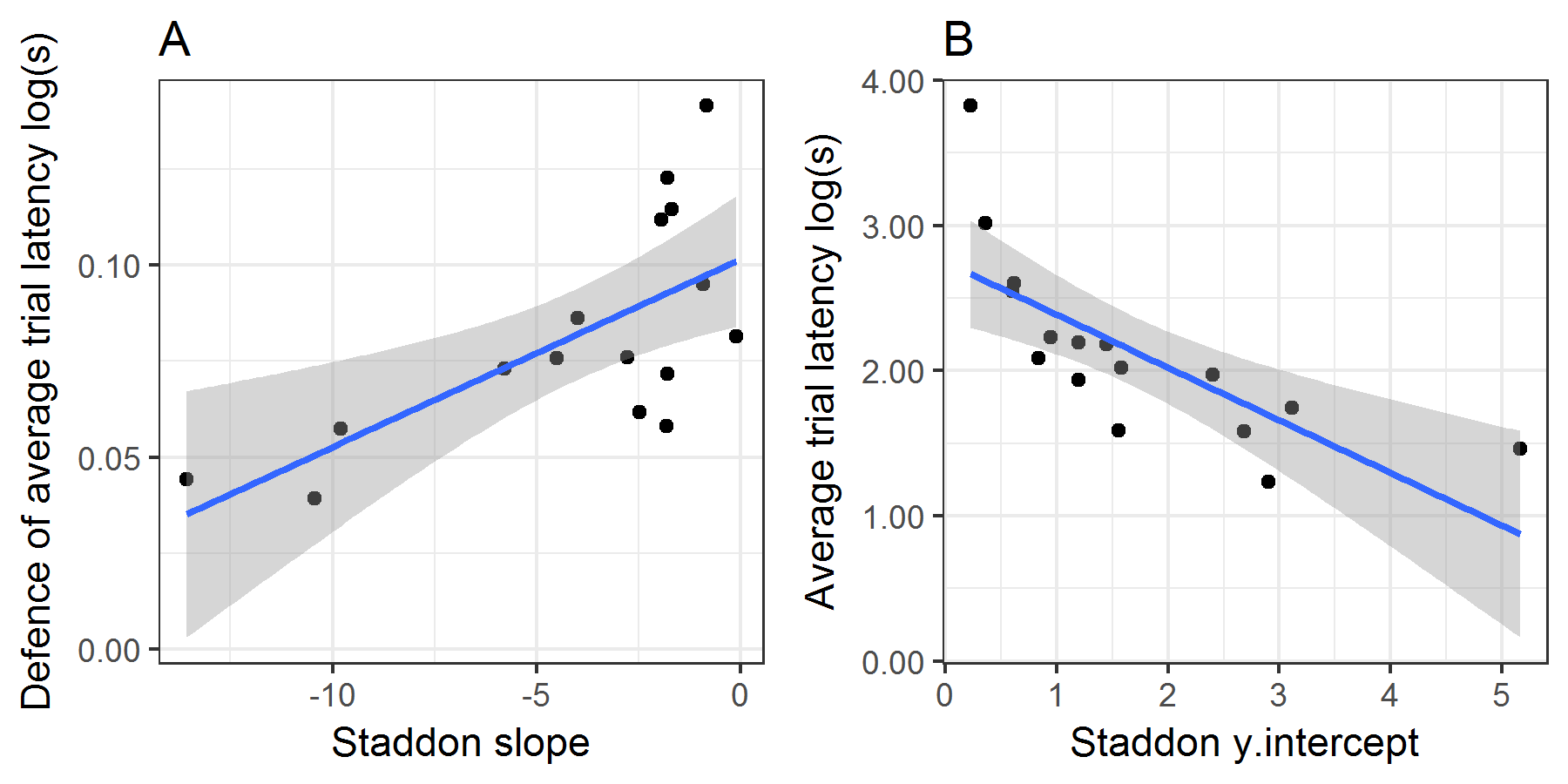


Figure S1. **Relationship between trial latencies and Staddon response function.** A) Defence of trial latency (slope of the relationship between ratio requirement and average trial latency) against Staddon response function slope, for each bird for whom the Staddon response function slope is defined. Note that here, unlike the figures in the main paper, defence is plotted so that a higher position on the y-axis represents weaker defence, to make the direction of the relationship with the Staddon measure clearer. Regression line represents a simple linear fit plus SE. B) Average trial latencies for individual birds against the Staddon y-intercept, for those birds for whom it is defined. Regression line represents a simple linear fit plus SE. Both panels are based on data from 16 birds.
